# Features of urinary *Escherichia coli* isolated from children with complicated and uncomplicated urinary tract infections in Mexico

**DOI:** 10.1371/journal.pone.0204934

**Published:** 2018-10-04

**Authors:** Víctor M. Luna-Pineda, Sara A. Ochoa, Ariadnna Cruz-Córdova, Vicenta Cázares-Domínguez, Juan P. Reyes-Grajeda, Marco A. Flores-Oropeza, José Arellano-Galindo, Rigoberto Castro-Hernández, Marcos Flores-Encarnación, Adriana Ramírez-Vargas, Héctor J. Flores-García, Leticia Moreno-Fierros, Juan Xicohtencatl-Cortes

**Affiliations:** 1 Laboratorio de Investigación en Bacteriología Intestinal, Hospital Infantil de México "Federico Gómez”, CDMX, México; 2 Laboratorio de Inmunidad en Mucosas, Unidad de Biomedicina, Facultad de Estudios Superiores Iztacala, Universidad Nacional Autónoma de México, Tlalnepantla, Estado de México, México; 3 Instituto Nacional de Medicina Genómica, CDMX, México; 4 Departamento de Infectología, Hospital Infantil de México "Federico Gómez”, CDMX, México; 5 Departamento de Ecología de Agentes Patógenos, Hospital General “Dr. Manuel Gea Gonzalez”, CDMX, México; 6 Facultad de Medicina, Benemérita Universidad Autónoma de Puebla, Puebla, México; 7 Facultad de Biología, Benemérita Universidad Autónoma de Puebla, Puebla, México; The Pennsylvania State University, UNITED STATES

## Abstract

The Hospital Infantil de México Federico Gómez (HIMFG) is a tertiary care hospital in Mexico City where *Escherichia coli* is frequently isolated from the urine samples of pediatric patients with urinary tract infections. A collection of 178 urinary *Escherichia coli* (UEc) isolates associated with complicated and uncomplicated urinary tract infections were evaluated in this study. The patterns of resistance to 9 antibiotic classes showed that 60.7% of the UEc isolates had a highly multidrug-resistant (MDR) profile. Genetic diversity analyses of the UEc isolates showed a high variability and revealed 16 clusters associated with four phylogenetic groups, namely, groups A, B1, B2, and D. Phylogenetic group B2 was widely associated with the 16 clusters as well as with virulence and fitness genes. The virulence and fitness genes in the UEc isolates, which included fimbriae-, siderophore-, toxin-, and mobility-associated genes, were grouped as occurring at a low, variable, or high frequency. Interestingly, only the *papF* gene could be amplified from some UEc isolates, and the sequence analysis of the *pap* operon identified an insertion sequence (IS) element and gene loss. These data suggested pathoadaptability and the development of immune system evasion, which was confirmed by the loss of P fimbriae-associated agglutination in the UEc isolates. *E*. *coli* clone O25-ST131 had a prevalence of 20.2% among the UEc isolates; these isolates displayed both a highly MDR profile and the presence of the *papG*II, *fimH*, *papG*III, *iutD*, *sat*, *hlyA*, and *motA* genes. In conclusion, the UEc isolates from complicated urinary tract infection (cUTI) were characterized as being MDR, highly genetically diverse, and associated with phylogenetic group B2 and many virulence and fitness genes. Additionally, gene loss and IS elements were identified in some UEc isolates identified as clone O25-ST131.

## Introduction

Urinary tract infections (UTIs) are the second leading cause of bacterial infections, and they affect approximately 150 million people worldwide per year. The following populations have a major risk of acquiring a UTI: newborns, preschoolers, sexually active women, and older individuals of both sexes [1]. A low incidence of UTIs has been associated with young men; however, prostatitis, asymptomatic bacteriuria, and diabetes-associated UTIs are considered of clinical importance [1]. UTIs are clinically classified as either uncomplicated or complicated. Uncomplicated UTIs (uUTIs) affect healthy individuals without structural or neurological abnormalities in the urinary tract; uUTIs are commonly known as cystitis (lower urinary tract) or pyelonephritis (upper urinary tract) and are mainly acquired in the community [2]. Complicated UTIs (cUTIs) have been associated with factors related to urinary tract dysfunction (urinary obstruction, urinary retention, and renal failure), host defense (immunosuppression, renal transplantation and pregnancy), and foreign bodies (calculi and catheters or other drainage devices) [3–6]. Catheter-associated UTI is considered the most important nosocomially acquired infection, raising the economic cost, increasing the number of hospital days per patient, and promoting the development of antibiotic resistance, thereby complicating treatment [7].

In Mexico, which sees approximately four million UTI cases per year, UTIs are a public health problem because of their high morbidity rate [8,9]. In recent years, 3,149,091 UTI cases have been reported in women of all ages; 1,392,235 of these infections occurred in women between 20 and 44 years of age. Furthermore, UTIs are the third leading cause of morbidity in males, with 957,875 cases per year. The incidence of UTIs has been associated with age; a decreased incidence of these infections is seen in adults over 44 years old. UTIs are the third leading cause of morbidity during puberty (15 to 19 years old), with 297,831 cases per year, as well as in pediatric patients (<15 years old; 360,220 cases per year) and children less than 1 year old (20,300 cases per year) [10]. In pediatric patients, the UTI incidence has been calculated to be 0.4–1.0% in girls, 0.18% in circumcised boys, and 0.7% in uncircumcised boys. In children (1 to 5 years old), the UTI incidence is 0.9–1.4% in girls and 0.1–0.2% in boys, while in children older than 6 years old, the incidence is 6.6% in girls and 1.8% in boys. In school-age children, the UTI incidence is 0.7–2.3% in girls and 0.04–0.2% in boys, while it is 3% in prepubertal girls and 1% in prepubertal boys [2,11]. UTIs in the first year of life are associated with anatomic or functional abnormalities; however, they are uncommon in boys of this age [12]. Young girls have multiple episodes of recurrent cystitis or pyelonephritis and display an increase in UTI incidence during adolescence [13].

Uropathogenic *Escherichia coli* (UPEC) is the most common causative agent of these infections [1]. Prior to the development of UTIs, UPEC colonizes its host through the expression of several virulence and fitness factors, such as flagella, capsules, siderophores, toxins, and adhesins [14]. The type 1 fimbrin D-mannose specific adhesin (FimH) protein of type 1 fimbriae, the major curlin subunit (CsgA) protein of curli, and variants of the fimbrial adhesin (PapG) protein of P fimbriae are the main fimbrial adhesins involved in the colonization of UPEC in the bladder. Recently, we reported that fusion proteins (designed from the FimH, CsgA, and PapG adhesins) are highly stable, have antigenic properties, and induce cytokine release and that antibodies against these proteins promote protection during bacterial infection [15]. UTI-associated UPEC clinical strains have been mainly classified into phylogenetic groups B2 and D and have shown a low frequency of association with phylogenetic groups A and B1 [16]. Similarly, we have reported that multidrug-resistant (MDR) and extensively drug-resistant (XDR) UPEC clinical strains collected from pediatric patients contain class I and II integrons and demonstrate an extended-spectrum β-lactamase (ESBL) phenotype [17]. Indeed, UPEC clone O25-ST131 has emerged as a main causative agent of UTIs and has been associated with ESBL and MDR profiles [18,19].

The Hospital Infantil de México “Federico Gómez” (HIMFG) is a tertiary care pediatric hospital in Mexico City. The institutional vigilance program tracking pathogens of clinical interest collected from blood, urine, and other sterile liquids has reported that *E*. *coli* is the second most frequently isolated pathogen of nosocomial (1772 children), community (3085 children), and unclassified (2851 children) origin [20]. The aim of this study was to evaluate the antibiotic resistance, virulence and fitness profiles and the molecular typification and phylogenetic distribution of a collection of urinary *E*. *coli* (UEc) isolates obtained from the HIMFG.

## Materials and methods

### Ethics statement

The study was reviewed and approved by the Research Committee (Dr. Juan Garduño Espinosa), Ethics Committee (Dr. Luis Jasso Gutiérrez), and Biosecurity Committee (Dr. Marcela Salazar García) of Hospital Infantil de México Federico Gómez (HIMFG), with permit numbers HIM/2016/099 SSA.1329, HIM/2017/002 SSA.1298, HIM/2017/136 SSA.1456 and HIM 2017–107 FF SSA.1421. Written informed consent was not required for this study according to the institutional ethical, biosecurity and investigation evaluation. The Central Laboratory of the HIMFG provided the UEc isolates from pediatric patients for this study.

### Bacterial isolates and control strains

From 2012 to 2013, a collection of 178 UEc isolates was obtained from pediatric patients with uUTIs and cUTIs in the HIMFG. The UEc isolates were selected according to an average bacterial count of ≥ 100,000 CFU/mL, as previously described [15,17]. In this study, the different experiments included control strains from the American Type Culture Collection (ATCC; VA, USA), as follows. The *E*. *coli* ATCC 25922 and *Pseudomonas aeruginosa* ATCC 27853 strains were used as controls for antimicrobial susceptibility testing. *E*. *coli* strains CFT073 (ATCC 700928) and J96 (ATCC 700336) were used as positive controls for the characterization of the virulence and fitness gene profiles. *E*. *coli* strains ECOR 60 (ATCC 35379), from group B2; ECOR 40 (ATCC 35359), from group D; ECOR 26 (ATCC 35345), from group B1; and ECOR 4 (ATCC 35323), from group A were used as positive controls for the identification of phylogenetic groups. The MDR UPEC clinical strain 42U-0612 was used as the identification control for the O25b-ST131 clone and was previously characterized by Ochoa et al. [17].

### Antimicrobial susceptibility of the UEc isolates

The microdilution method was used to determine the antimicrobial susceptibility profile of the 178 UEc isolates according to the 2016 Clinical and Laboratory Standards Institute guidelines. The minimum inhibitory concentration (MIC) was determined for ampicillin (AM; Sigma Aldrich, MO, USA), amoxicillin-clavulanate (AMC; Glaxo Smith-Kline, Brentford, Middlesex, UK), piperacillin-tazobactam (TZP; Siemens Medical Solutions USA, Inc., PA, USA), cephalothin (CF; Eli Lilly and Company; IN, USA), cefaclor (CEC; Thermo Scientific, MA, USA), ceftazidime (CAZ; Sagent Pharmaceuticals, Inc., IL, USA), norfloxacin (NOR), ofloxacin (OFX; MP Biomedicals, OH, USA), meropenem (MEM), imipenem (IPM; AstraZeneca Pharmaceuticals LP, DE, USA), gentamycin (GM; Schering-Plough Pharmaceuticals, NJ, USA), trimethoprim-sulfamethoxazole (SXT; Roche, Basel, Switzerland), and nitrofurantoin (F/M; McKesson Pharmaceutical, CA, USA). MDR strains were defined as those that were not susceptible to at least one antibiotic in three or more classes.

### Enterobacterial repetitive intergenic consensus-PCR analysis of the UEc isolates

Molecular typing of the UEc isolates was performed using enterobacterial repetitive intergenic consensus polymerase chain reaction (ERIC-PCR) according to specific modifications of the protocols established by Ardakani and Ranjbar [21]. ERIC-PCR was performed on 25 μL reaction volumes containing 1 μL of each primer (25 pmol), 12.5 μL of the master mix, 1.5 μL of MgCl_2_ (300 nM), 1 μL of the DNA template (100 ng/μL), and 8 μL of deionized water. The ERIC-PCR protocol was divided into 3 steps: 1) denaturation at 94°C for 2 min; 2) 30 cycles of denaturation at 94°C for 1 min, annealing at 52°C for 1 min, and extension at 72°C for 4 min; and 3) a final extension at 72°C for 5 min. PCR products were resolved on 2% agarose gels (Promega Corporation, WI, USA) by electrophoresis at 80 volts for 120 min. The PCR products resolved on the gels were stained with 0.5 mg/mL ethidium bromide (Sigma Aldrich) for 40 min and visualized under UV light using a ChemiDoc MP imaging system. A 1 kb ladder and a 100 bp ladder (Promega Corporation; WI, USA) were used as molecular weight markers.

### Phylogenetic grouping of the UEc isolates

One hundred seventy-eight UEc strains were cultured in Luria-Bertani (LB; Difco-Becton Dickinson; NJ, USA) broth, and genomic DNA was extracted using a Wizard^®^ Genomic DNA Purification kit (Promega Corporation, WI, USA) according to the manufacturer’ instructions. The amplification of *chuA*, *yjaA*, and one genomic fragment (TspE4.C2) was performed on the extracted DNA by multiplex PCR using specific primers (S1 Table). The PCR products were resolved by electrophoresis on 1.5% agarose gels (Promega Corporation, WI, USA), stained with ethidium bromide solution (Sigma-Aldrich, St. Louis, MO, USA), and visualized with a gel imaging system (ChemiDoc MP System, Bio-Rad; Mexico). The phylogenetic groups were assigned according to the following genotypes: group B2 (*chuA*^+^/*yjaA*^+^), group D (*chuA*^+^/*yjaA*^*−*^), group B1 (*chuA*^*−*^/TspE4.C2^+^), and group A (*chuA*^*−*^/TspE4.C2^*−*^) [16].

### Identification of clone O25b-ST131 in the UEc isolates

Allele-specific PCR was used to amplify the 347 bp aminodeoxychorismate synthase component 1 (*pabB*) gene using specific primers (S1 Table). The amplification of this gene has been used to identify the *E*. *coli* isolate clone O25b-ST131. The DNA quality was evaluated by amplifying a 427 bp fragment corresponding to the tryptophan synthase alpha chain (*trpA*) gene. The PCR conditions and specific primers were described by Clermont et al. [22]. PCR was performed with PCR MasterMix (Promega Corporation, WI, USA) and 2 μL of purified DNA in a total reaction volume of 20 μL under the following conditions: denaturation at 95°C for 4 min; 30 cycles of denaturation at 94°C for 30 sec, annealing at 65°C for 10 sec, and extension at 72°C for 30 sec; and a final extension at 72°C for 5 min.

### Identification of virulence and fitness genes in the UEc isolates

The *papG* variants (I, II and III), *fimH* and *csgA* fimbriae-associated genes contained in the 178 UEc isolates were identified by multiplex PCR using specific primers (S1 Table). Additionally, the P fimbrial adapter (*papF*) gene was amplified and used for the identification of new *papG* variants in the isolates testing negative for the canonical *papG* variants. Similarly, the yersiniabactin (*fyuA*) and aerobactin (*iutD*) siderophore-associated genes as well as the alpha-hemolysin (*hlyA*), secreted autotransporter toxin (*sat*), and type 1 secretion A (tosA) toxin-associated genes were identified.

### Evaluation of *pap* loci

The UEc isolates were evaluated for the absence of *papG* variants and the presence of the *papF* gene by PCR using specific primers; additionally, deletion and insertion mutations in the *papG* locus were evaluated by DNA sequencing (S1 Table). The amplifications were performed with a high-fidelity *Pfu* polymerase from Thermo-Fisher Scientific (CA, USA), and the products were cleaned and concentrated using a Zymo DNA Clean and Concentrator kit from Zymo Research (CA, USA). The amplicons were cloned into a pJET-Blunt 1.2 vector (Thermo-Fisher Scientific, CA, USA) and were subjected to sequencing on a 3730*xl* DNA Analyzer from Applied Biosystems^™^ (CA, USA) at the “Unidad de Secuenciación-Instituto Nacional de Medicina Genómica” (CDMX, Mexico). The obtained sequences were analyzed using the ClustalO (https://www.ebi.ac.uk/Tools/msa/clustalo/) and Basic Local Alignment Search Tool (BLAST) algorithms from the National Center of Biotechnology Information (NCBI). PCR was used with specific primers to identify the presence of other insertion sequences (IS) in the *pap* loci and to amplify all *pap* genes (*papI* to *papG*) as well as the following internal regions: *papI* to *papE*, *papI* to *papA*, and *papA* to *papE*.

### Graphics

Heat maps were generated with XLSTAT software from Addinsoft SARL (NY, USA). ERIC-PCR analysis was performed using NTSYS-pc software (version 2.0, Applied Biostatistics, Inc., NY, USA).

## Results

### Source and antibiotic resistance profiles of the UEc isolates

The 178 UEc isolates were collected from patients with UTIs in different wards of the HIMFG. Briefly, 67.4% (120/178) of the isolates were collected from patients in the nephrology (NP), urology (UR), pediatric intensive care unit (PICU), neonatal intensive care unit (NICU), oncology (ONC), hematology (HEM), general pediatric surgery (GPS), transplant unit (TRU), tissue engineering (TE), internal therapy (IT), surgical therapy (ST), infectology (INF), internal medicine (IM), and emergency (EW) wards; these isolates were considered cUTIs (S2 Table). The 32.6% (58/178) of the UEc isolates from patients without structural or neurological urinary tract abnormalities were considered uUTIs; these isolates were obtained from patients in the classification (CLW), gastroenterology (GA), children’s stay (CS), pediatrics (PED), adolescent (AD), allergy (AL), external consultation (EC), and neurology (NEU) wards (S3 Table). The resistance profile for the 9 classes of antibiotics showed the following distribution: 14.3% (26/178) of the UEc isolates were sensitive to all tested antibiotics, 5.6% (10/178) to one class of antibiotics, and 19.1% (34/178) to two classes of antibiotics. Interestingly, 60.7% (108/178) of the UEc isolates had an MDR profile; among them, 31.5% (34/108) were resistant to three antibiotic classes, 9.3% (10/108) to four classes, 8.3% (9/108) to five classes, 28.7% (31/108) to six classes, 16.7% (18/108) to seven classes, and 5.6% (6/108) to eight classes. The source of UEc isolates and their antibiotic resistance profiles are described in S2 and S3 Tables.

### Genetic diversity and phylogenetic grouping of the UEc isolates

**Fig 1:**
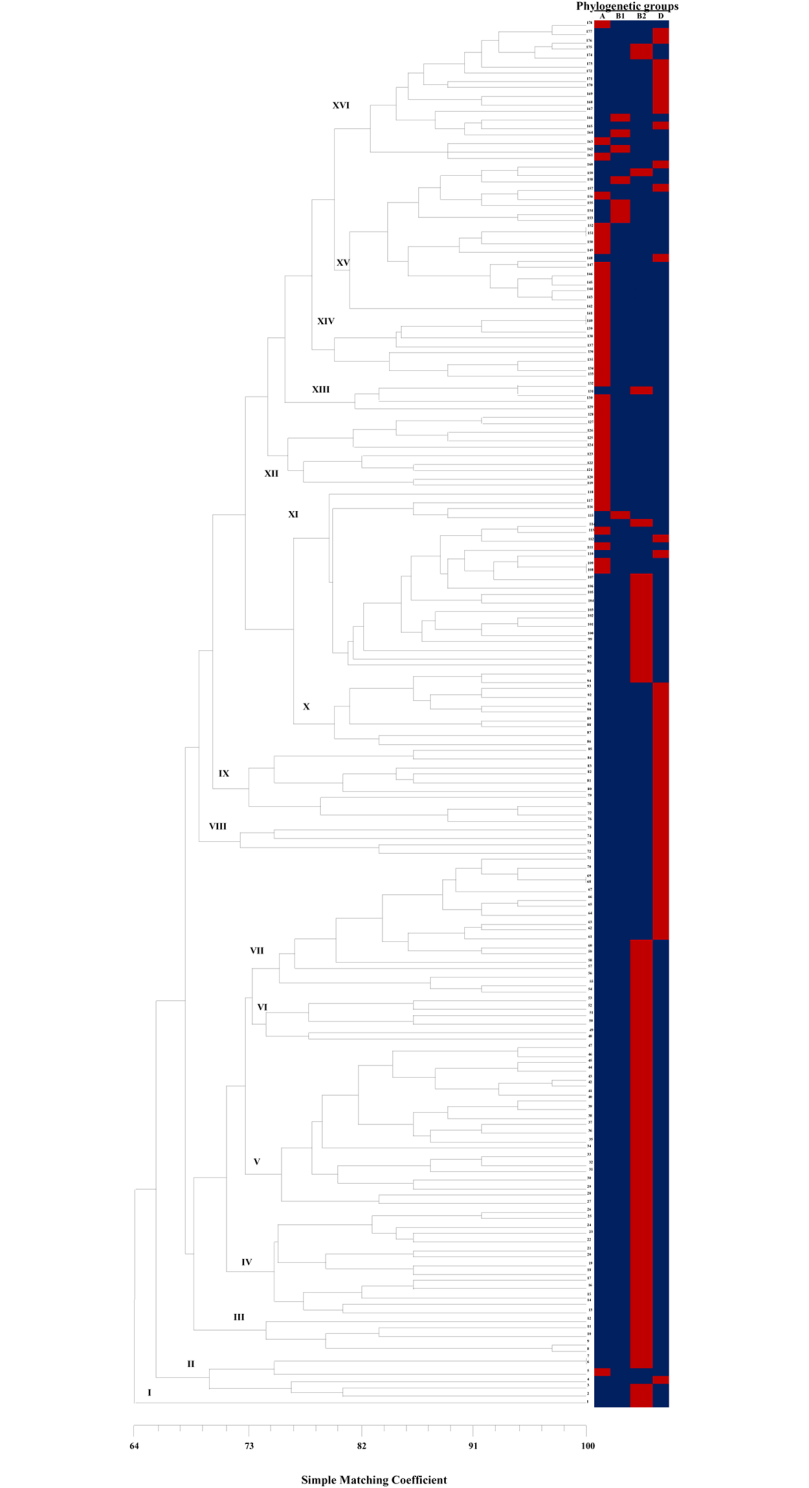
ERIC-PCR analysis of UEc isolates associated with phylogenetic groups. The dendrogram generated from the ERIC-PCR analysis of 178 UEc isolates revealed 16 clusters. Additionally, the dendrogram was evaluated using the cophenetic correlation coefficient obtained by the Mantel test, which indicated the dispersion of the data and had a value of r = 0.80. An association with phylogenetic groups is shown in the dendrogram. The color map shows positive results in red and negative results in blue.

The ERIC-PCR results revealed 5–10 DNA fragments ranging in size from 250 to 2,500 bp (Fig 1). A total of 5.6% of the isolates were grouped between UEc-6 and -7, UEc-68 and -69, UEc-108 and -109, and UEc-140 and -141, which were isolated from the same patient. Genetic diversity analyses of the UEc isolates showed the presence of sixteen clusters: 0.6% (1/178) of the isolates were distributed into cluster I, 3.4% (6/178) into cluster II, 2.8% (5/178) into cluster III, 7.9% (14/178) into cluster IV, 11.8% (14/178) into cluster V, 3.4% (6/178) into cluster VI, 10.1% (18/178) into cluster VII, 2.2% (4/178) into cluster VIII, 5.6% (10/178) into cluster IX, 5.6% (10/178) into cluster X, 12.9% (23/178) into cluster XI, 5.6% (10/178) into cluster XII, 2.2% (4/178) into cluster XIII, 5.1% (9/178) into cluster XIV, 10.7% (19/178) into cluster XV, and 10.1% (18/178) into cluster XVI (Fig 1).

However, the UEc isolates were distributed into four phylogenetic groups: 43.2% (77/178) into group B2, 27.5% (49/178) into group D, 24.8% (44/178) into group A and 8.0% (8/178) into group B1. Group A was associated with four clusters (I, V, VI, and VII), group B1 was associated with one cluster (IV), group B2 was associated with seven clusters (VIII, IX, XII, XIII, XIV, XV and XVI), and group D was associated with four clusters (II, III, X, and XI) (Fig 1). Interestingly, 100% of the MDR UEc isolates belonging to the B2 group were included in clusters XIV and XV.

### Distribution of virulence and fitness genes in the UEc isolates

**Fig 2:**
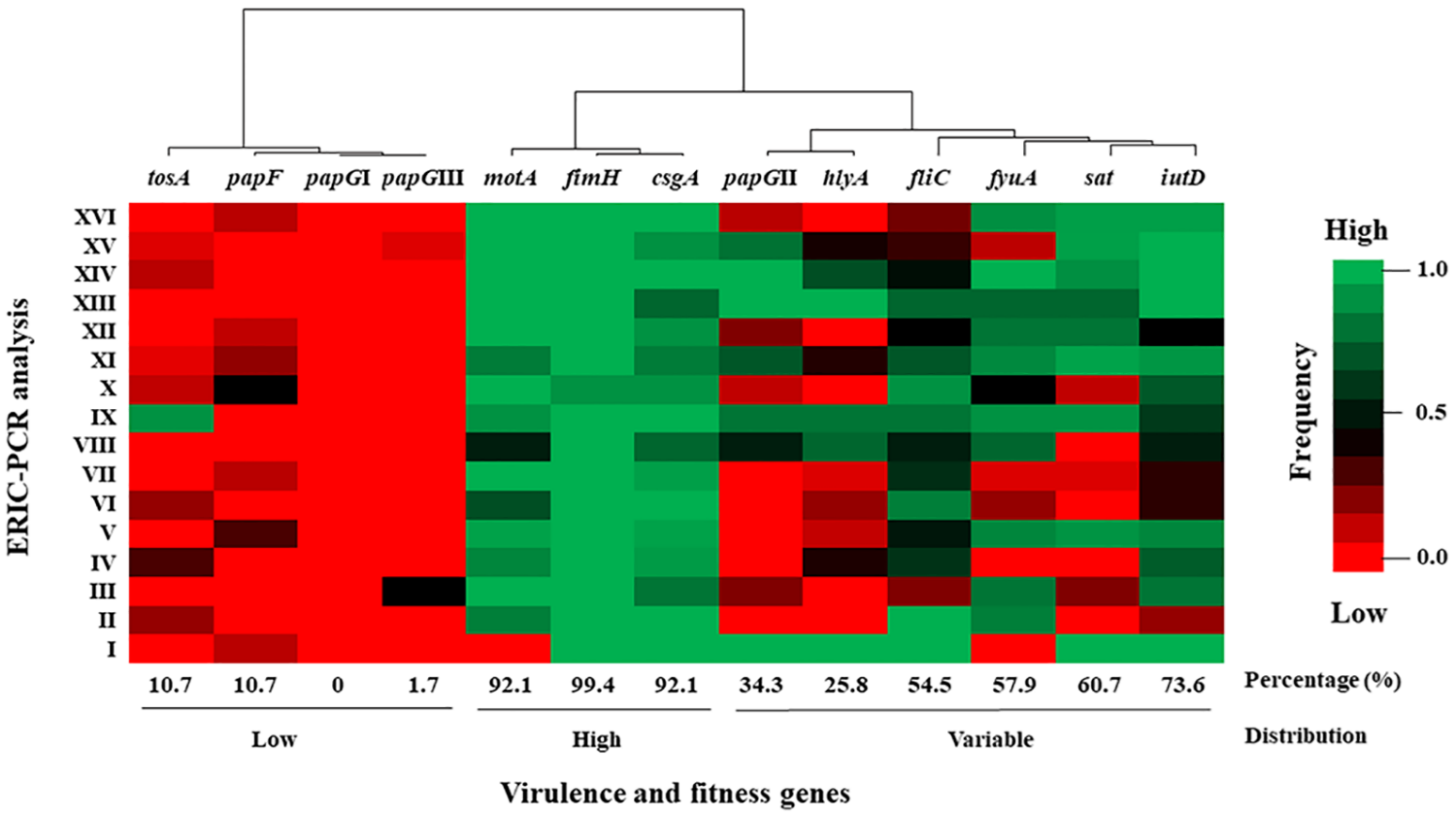
Distribution of virulence and fitness genes associated with the genetic diversity of the UEc isolates. Heat map showing the distribution of virulence and fitness genes, including genes associated with fimbria, mobility, toxins, and iron uptake, in UEc isolate strains. The virulence and fitness genes were grouped as being present in UEc isolates at a low, variable or high frequency and were associated with the 16 clusters obtained from the ERIC-PCR analysis. The percentages of virulence and fitness genes are shown below the figure, and the frequency scale is shown to the right of the figure.

The main virulence and fitness genes in the UEc isolates were distributed in the following order: 99.4% (177/178) of the isolates contained *fimH*; 92.1% (164/178), *csgA*; 92.1% (164/178), *motA*; 73.6% (131/178), *iutD*; 60.7% (108/178), *sat*; 57.9% (103/178), *fyuA*; 54.5% (97/178), *fliC*; 25.8% (46/178), *hlyA*; 10.7% (19/178), *tosA*; 34.3% (61/178), *papG*II; 1.7% (3/178), *papG*III; and 10.7% (19/178), *papF* (Fig 2). However, the *papG*I gene was not identified in any UEc strain (Fig 2).

**Fig 3:**
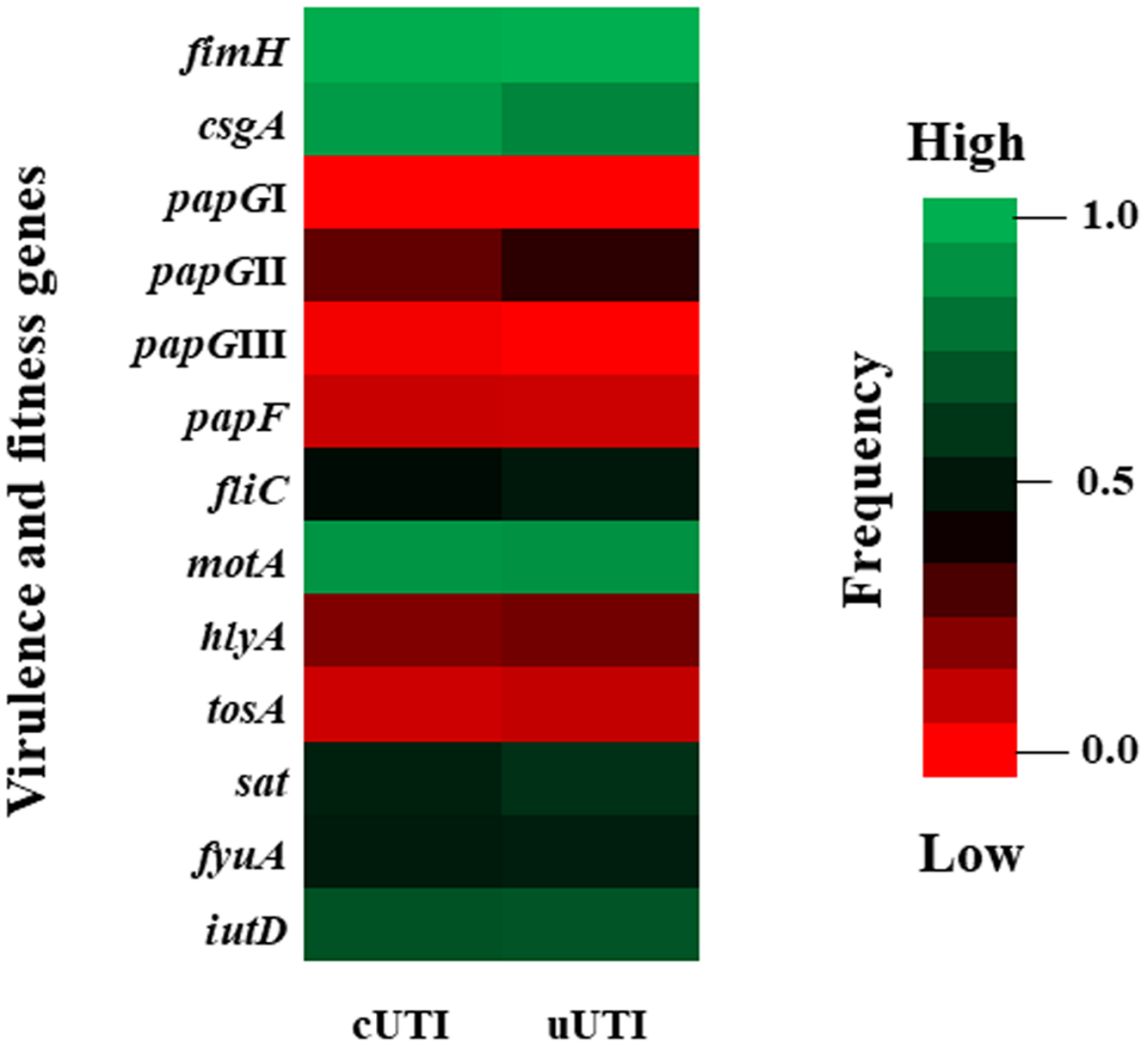
Distribution of virulence and fitness genes associated with the clinical classification of the UEc isolates. Heat map showing the distribution of virulence and fitness genes in UEc isolate strains, including genes associated with fimbria, mobility, toxins, and iron uptake. Clinically, these isolates were grouped as cUTIs and uUTIs, and the gene frequencies were compared between these groups. The frequency scale is shown to the right of the figure.

According to the genetic diversity analysis, the *fimH*, *csgA*, and *motA* genes were present at a high frequency in all previously described clusters; however, the *motA* gene was not identified in cluster I (Fig 2). The *fliC*, *hlyA*, *sat*, *fyuA* and *iutD* genes were present at a variable frequency in the 16 clusters; nevertheless, the *hlyA* gene had the highest association with clusters I and XIII. Interestingly, the *sat* gene was present at a low frequency in three clusters (III, VII and XIV) and was absent in four clusters (II, IV, VI and VIII); additionally, the *fyuA* gene was absent in clusters I and IV. The essential genes (*papG*II and III; *papF*) that code for proteins involved in P fimbriae assembly, along with the *tosA* gene, were present at a low frequency. Furthermore, the *tosA* gene was mainly associated with cluster IX (Fig 2). Clinically, UEc isolates were classified as cUTI or uUTI. The highest frequency of virulence and fitness genes was associated with the uUTIs isolates, including *iutD*, *csgA*, *sat*, *tosA*, *fliC* and *papG*II, while the frequency of *fimH* was observed to be lower than that of gene from cUTI isolates (Fig 3). Interestingly, the highest frequency of the *papG*III gene was described in these isolates (Fig 3).

**Fig 4:**
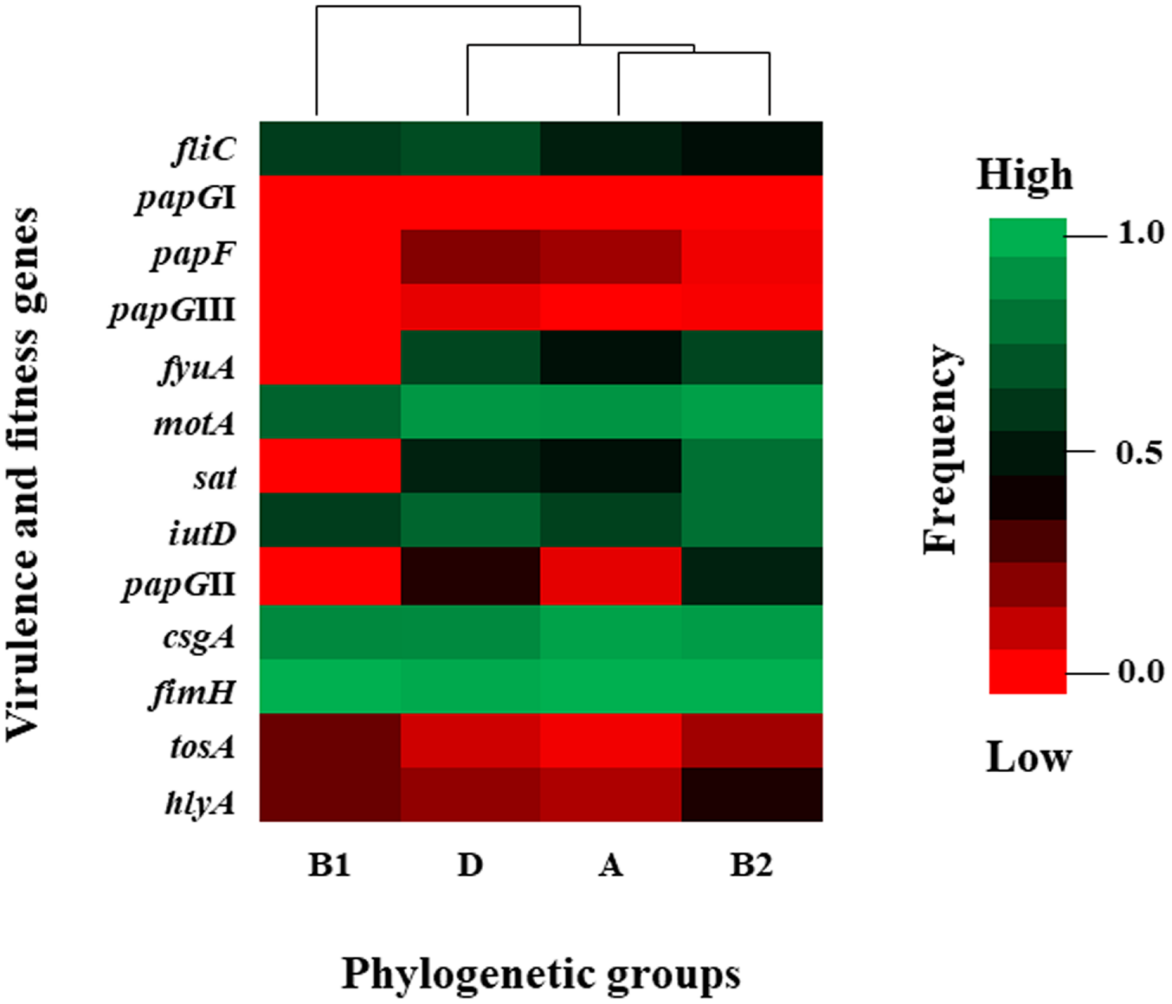
Distribution of virulence and fitness genes associated with the phylogenetic groups. Heat map showing the distribution of virulence and fitness genes, including genes associated with fimbria, mobility, toxins, and iron uptake, in UEc isolate strains. The virulence and fitness genes were associated with phylogenetic groups A, B1, B2 and D. The frequency scale is shown to the right of the figure.

However, virulence- and fitness-associated genes, including those coding for fimbrial adhesins and factors associated with mobility, toxins, and iron uptake, were mainly associated with phylogenetic groups D and B2. The *papG*II variant was associated with phylogenetic groups D and B2 with frequencies of 0.36 and 0.53, respectively, and it was associated with phylogenetic group A with a frequency of 0.04. The *papG*III was associated with phylogenetic groups B2 and D with frequencies of 0.012 and 0.040, respectively. Three virulence genes (*sat*, *fyuA*, and *pap*) were absent from phylogenetic group B1. The *hlyA*, *tosA*, *iutD*, and *fliC* genes were associated with phylogenetic group B1 with high frequencies ranging from 0.25 to 0.62 (Fig 4).

The *papF* gene, which was amplified to verify the absence of the *pap* operon, was present in phylogenetic groups D and A at frequencies of 0.15 and 0.20, respectively (Fig 3). Interestingly, *pap*F was amplified from 10.7% (19/178) of the UEc isolates, and amplicons of ~2,500 bp were generated for 47% (9/19) of them using specific primers designed to amplify a region encompassing 100 bp upstream and downstream of the *papG* open reading frame (ORF); however, under the same conditions, a final product of ~1,400 bp was obtained for the *pap*G gene of strain CFT073.

### Deletion and insertion mutations in the *pap* loci

**Fig 5:**
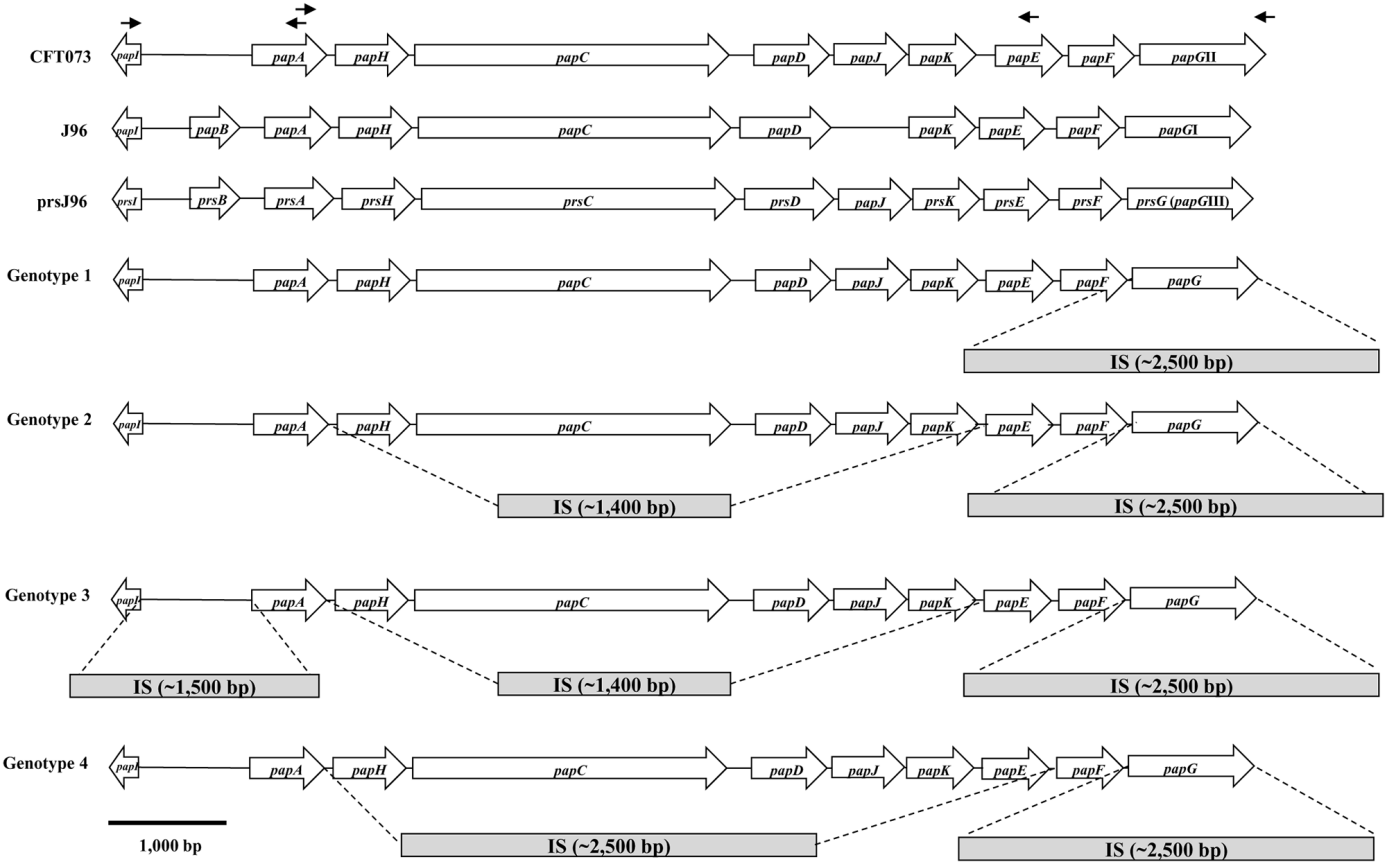
Analysis of the *pap* operon. PCR amplification and DNA sequencing showed IS elements and gene loss in the *pap* operon of some UEc isolates. The specific primers used are represented as arrows and are listed in S1 Table. Three sequences, namely, the CFT073 (AE014075.1) *pap* operon and the J96 (NZ_ALIN02000070.1) *pap* and *prs* operons, were used as control strains. The IS elements are represented by squares, and the regions representing the probable insertions are indicated by dashed lines. The solid line represents a 1,000 bp scale bar.

The DNA sequencing of ~2,500 bp amplicons followed by sequence bioinformatics analysis showed an IS element between *papX* and *papF*, which contained a *papG* pseudogene followed by a putative IS3 family transposase gene along with three genes encoding hypothetical proteins. This IS element showed homology (98% identity) with sequences from *E*. *coli* strains such as YDC107 (GenBank accession no. CP025707.1, *locus* 1,625,045 to 1,628,120), CE10 (GenBank accession no. CP003034.1, *locus* 3,492,275 to 3,494,785), and IAI39 (GenBank accession no. CU928164.2, *locus* 3,549,993 to 3,553,055). PCR analysis of the *pap* operon for the UPEC strains J96 (containing *papG*I; ALIN02000070.1 and *prsG* [variant III]; X61239.1) and CFT073 (*papG*II; AE014075.1) showed amplicons of ~7,000 bp (*papI* to *papE*), ~1,200 bp (*papI* to *papA*), ~8,000 bp (*papI* to *papG*), and ~5,800 bp (*papA* to *papE*) (Fig 5). The amplifications obtained from nine previously described UEc isolates showed four isolates that contained only the ~2,500-bp IS element and were classified as genotype 1. Genotype 2 was identified in three UEc isolates and showed amplicons of ~2,500 bp for *papI* to *papE* and ~1,200 bp for *papI* to *papA*. An IS element of ~1,400 bp in this genotype was likely related to the absence of five genes (*papH*, *papC*, *papD*, *papJ* and *papK*). The IS elements of ~2,500 bp and ~1,400 bp were identified in one isolate with a new amplicon of 2,500 bp for *papI* to *papA*, while the *pap* operon was classified as genotype 3 and probably acquired another IS element of ~1,500 bp in size. Genotype 4 was described in one UEc isolate showing the ~2,500 bp IS element; however, amplicons of ~5,500 bp for *papI* to *papG* and ~1,200 bp for *papI* to *papA* were identified. An IS element of ~2,500 bp in this genotype was likely related to the absence of six genes, namely, *papH*, *papC*, *papD*, *papJ*, *papK*, and *papE* (Fig 5). Finally, the agglutination phenotype was evaluated in these isolates and was compared with the UPEC strain CFT073, which showed a loss of agglutination (data not shown).

### Characteristics of the O25b-ST131 clone in the UEc strains

**Fig 6:**
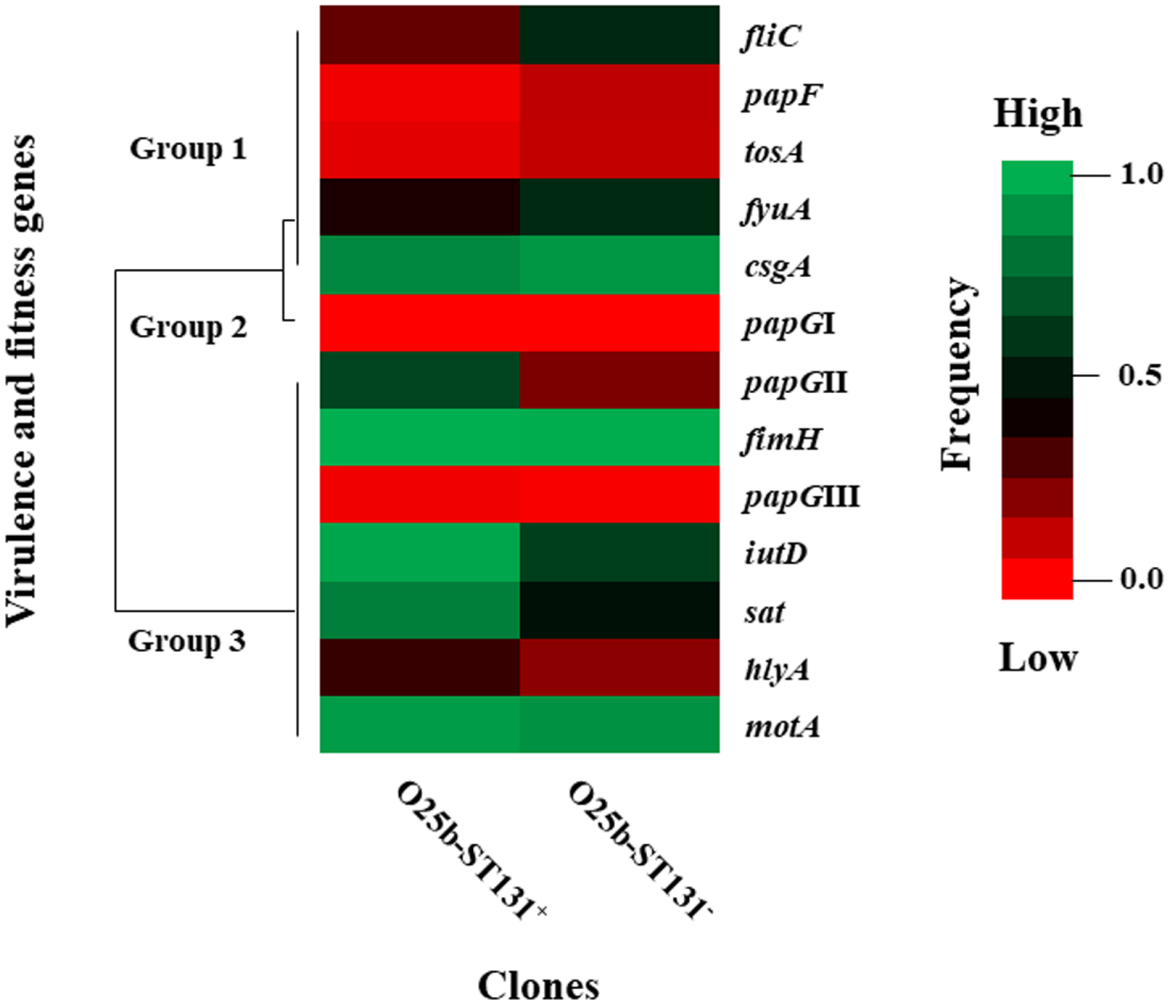
Distribution of virulence and fitness genes associated with clone O25b-ST131 UEc isolates. Heat map showing the distribution of virulence and fitness genes, including genes associated with fimbria, mobility, toxins, and iron uptake, in UEc isolate strains. The virulence and fitness genes in the UEc isolates were associated with clone O25b-ST131 according to the following three groups: low, variable and high frequency. The frequency scale is shown to the right of the figure.

Based on the high frequency of MDR (60.7%) in the UEc isolates, we searched the prevalence of the clone O25-ST131, which has been associated with UPEC strains with ESBL CTX-M-15 belonging to phylogenetic group B2, carrying several virulence and fitness genes. The amplification of the *pabB* gene was identified in 20.2% (36/178) of the UEc isolates considered to be clones of O25b-ST131. Of these isolates, 91.6% (33/36) demonstrated an MDR profile with the following resistance patterns: 6.06% (2/33) were resistant to three groups of antibiotics, 3.03% (1/33) to four groups, 3.03% (1/33) to five groups, 57.5% (19/33) to six groups, 27.2% (9/33) to seven groups, and 3.03% (1/33) to eight groups. The O25b-ST131 clones were distributed into phylogenetic groups B2 and A at frequencies of 88.9% (32/36) and 11.1% (4/36), respectively. The gene distribution in the O25b-ST131 clones was classified into three groups: the *fliC*, *papF*, *tosA*, *fyuA*, and *csgA* genes were classified into group 1; however, none of these genes were present at a high frequency in the O25b-ST131 clones. The *papG*I gene was classified into group 2 and was absent in all the UEc isolates (Fig 6). The *papG*II, *fimH*, *papG*III, *iutD*, *sat*, *hlyA* and *motA* genes were classified into group 3 and were present at a high frequency in the O25b-ST131 clones; interestingly, these genes were present at a lower frequency in the isolates that were not O25b-ST131 clones (Fig 6).

## Discussion

The HIMFG is a tertiary care hospital in Mexico City mainly focused on attending to pediatric patients with neoplasias such as leukemia and lymphoma. During their hospital stay, the children receive chemotherapy, which results in neutropenia and immunodeficiency-associated infections. *E*. *coli* is the second most common bacteria isolated from pediatric patients at the HIMFG; 74.6% of the affected patients are hospitalized, and 25.4% are outpatients. Eighty percent of the *E*. *coli* isolates were isolated from the urine of patients with a uUTI or a cUTI. In this study, 178 UEc isolates were evaluated; of these, 32.6% were obtained from patients with uUTIs, and 67.4% were obtained from patients with cUTIs. According to the antibiotic resistance profiles, 60.7% of the UEc isolates included in this study demonstrated an MDR profile. Our data showed a higher percentage of MDR isolates than that observed in another systematic review and meta-analysis of MDR UPEC clinical strains, which estimated the percentage to be 49.4% [23]. Recently, our group showed that 20.6% of MDR UPEC clinical strains were isolated from pediatric patients with cUTIs [17].

Our genetic diversity analysis of the UEc isolates showed a high variability; 16 clusters (I-XVI) were generated without identifying an association with the origin and antibiotic resistance profiles. The 178 UEc isolates were distributed into four phylogenetic groups, and the phylogenetic group B2 was distributed into 16 clusters. ERIC-PCR has been used for tracing the transmission of bacteria such as *Pseudomonas*, *Haemophilus*, *Vibrio*, *Corynebacterium*, *Salmonella*, and *Streptococcus*, in the community and in hospitals [24–29]. Other studies have identified UPEC strains mainly associated with phylogenetic group B2 in Mexican women and men with UTIs [30,31]. Interestingly, phylogenetic groups A and B1 have been related to commensal *E*. *coli* strains with the ability to cause several infections associated with diarrheagenic *E*. *coli*; however, these phylogenetic groups have also been identified in uUTIs and cUTIs [32]. UPEC strains related to phylogenetic groups B2 and D are associated with the geographical area of origin, the infection site, antibiotic resistance, and virulence [33,34]. The phylogenetic distribution of UEc could be considered to be predictive of the isolates present in pediatric patients with UTIs. In addition, this study sought to identify virulence and fitness genes associated with fimbria, mobility, toxins, and iron uptake present in the UEc isolates. In the UEc isolates, these genes were classified as having a low, variable, or high frequency. The genome core-localized genes, such as *motA*, *fimH*, and *csgA*, were identified in all UEc strains. An example of adaptation in extraintestinal *E*. *coli* is the positive selection of specific residues in FimH that enhance adherence to the urinary tract [35]. New data generated by our group suggest that the CsgA protein, which is the major subunit of curli, might use the same adaptation strategy as FimH (manuscript in preparation). Other studies have identified similar isolation frequencies among UPEC isolates from patients with cystitis, pyelonephritis and bacteremia [36,37]. The pathogenicity island (PAI)-localized genes (*papG*II, *hlyA*, *fyuA*, *sat* and *iutD*) have been acquired by horizontal transfer and were identified at variable frequencies in the UEc isolates. These genes could have a variable frequency of acquisition through PAIs, fitness islands and/or plasmids [38,39]. In addition, whole-genome sequencing of 14 UPEC strains showed multiple genetic origins with different acquired genes [40]. Compared with the *motA* gene, which codes for a structural protein of the flagellar system and is considered to have a high distribution, the *fliC* gene, which is localized in the genome core, was identified at a variable frequency in the UEc isolates. Horizontal transfer and intragenic recombination have promoted heterogeneity in divergence between the sequences of the *fliC* gene; therefore, this variation in the gene sequence could generate false negatives when conventional analysis methods are used. The *papF*, *papG*I, *papG*II, and *tosA* genes were present at a low frequency in the UEc isolates included in this study; this finding corresponded with those of other studies [33,34]. Interestingly, the *tosA* gene identified in cluster IX showed the highest frequency of virulence- and fitness-associated genes. The presence of the *tosA* gene has been associated with UPEC clinical strains carrying an unusually high number of virulence and fitness genes [41]. UEc isolates carrying the *tosA* gene belonging to phylogenetic groups B1 and B2 were more common than those belonging to groups A and D; therefore, UPEC strains associated with group B1 acquired *tosA* by horizontal transfer from phylogenetic group B2 [42]. UEc isolates associated with phylogenetic groups A and B1 are considered commensal *E*. *coli*, and virulence and fitness genes were present at a low frequency in phylogenetic group B1. The *tosA* gene associated with phylogenetic group B1 was likely acquired by horizontal transfer into a genomic island, thus allowing commensal *E*. *coli* to successfully colonize the urinary tract [41]. Phylogenetic group A was related to group B2, suggesting that the acquisition of virulence and fitness genes (*hlyA*, *sat*, *fyuA*, *iutD*, and *papG*) present in these isolates occurred by horizontal transfer. The *pheV* island of UPEC strain CFT073, which carries the *hlyA*, *pap*, *iha*, *sat*, *iut*A, *iucDCBA*, antigen 43 precursor and *kpsTM* genes, could be transferred to commensal strains [43]. UEc isolates from uUTI showed a higher frequency of virulence and fitness genes than did those from cUTI. Similar data were described in *E*. *coli* isolates from uncomplicated and complicated cystitis [44].

Variants of the *papG* gene were not amplified in some UEc isolates; however, the *papF* gene was identified in these isolates and was associated mainly with phylogenetic groups A and D. These data suggested probable modifications in the sequence of the *papG* gene; the modifications could be point mutations, deletions and/or insertions, as described in both *csg* operons of *Shigella* [45]. The amplification of the region encompassing 100 bp upstream and downstream of the *papG* ORF resulted in an amplicon of ~2500 bp that contained the IS element and the *papG* pseudogene. An assessment of the *papG* sequence in a collection of UPEC strains showed amplicons of ~1,100 bp for *papG*II and III; however, a *papG*I amplicon of ~1,200 bp and a new *papG* variant termed *papG*IV were also identified [46]. Our data showed the presence of these insertions in the *papG* gene of nine of the UEc isolates. However, in other studies, the ~2500 bp amplicon of the *papG* gene was not identified. Interestingly, the sequence of the ~2500 bp amplicon showed homology to the *pap locus* of the YDC107, CE10, and IAI39 *E*. *coli* strains, which are associated with bacteremia, neonatal meningitis, and pyelonephritis, respectively [47–49]. Other IS elements have been reported in the *pap* operon of *E*. *coli* strains, including the nonpathogenic probiotic *E*. *coli* Nissle 1917 strain [50]. An evaluation of the *pheV* genomic island in the IAI39 UPEC strain showed gene acquisition and loss affecting genes including *pap*-associated genes and the *hly* operon [49]. UEc isolates producing ~2500 bp amplicons of *papG* did not contain the *hlyA* gene, indicating that these isolates probably lost the *hly* operon, as reported for the IAI39 UPEC strain. *Shigella* spp. and enteroinvasive *E*. *coli* strains contained insertions or deletions in the *csg* locus or were unable to produce curli, suggesting a pathoadaptive mutation and evasion of the immune system [45,51]. Pathoadaptive mutations in UPEC have been reported to induce variation of FimH of type 1 fimbriae and PapG of P fimbriae; however, the loss of genes by the insertion of IS elements into the *pap* operon has not been reported in UPEC [52].

*E*. *coli* clone O25-ST131 has emerged worldwide and is associated with community-acquired and antimicrobial-resistant UTIs [53]. The geographical distribution of clone O25-ST131 has been reported; this clone has a high prevalence in the UK (81% in 2004–2007), the Central African Republic (50% in 2004–2006), France (46% in 2006–2007) and Canada (41% in 2004–2006). A low prevalence of clone O25-ST131 was reported in Cambodia (27% in 2004–2005), Turkey (20% in 2006), and Mexico (17% in 2017) [19,22,53]. In this study, the UEc isolates showed a prevalence of 20.2% for clone O25b-ST131, and this clone was strongly associated with the MDR profile. Interestingly, 88.9% of the clones O25-ST131 were resistant to fluoroquinolone. These data indicate a higher prevalence of this clone than do previous reports, which indicate that 30 to 60% of *E*. *coli* clones O25b-ST131 are fluoroquinolone-resistant [19]. A decrease in the presence and/or expression of some virulence factors has been associated with quinolone resistance in strains belonging to phylogenetic group B2 [54]. However, the UEc isolates of clone O25-ST131 were distributed into phylogenetic groups B2 and A and contained many virulence and fitness genes, such as *papG*II, *fimH*, *papG*III, *iutD*, *sat*, *hlyA*, and *motA*. In addition, *E*. *coli* clone O25-ST131 has been associated with an MDR strain producing the ESBL CTX-M-15 that carries several virulence genes and is mainly related to phylogenetic group B2, as described in this study [55].

## Conclusions

UEc strains isolated from Mexican children in the HIMFG primarily displayed MDR profiles and high genetic diversity. Core genome-associated virulence and fitness genes were more widely distributed than genes associated with PAIs, which were found mainly in isolates classified in uUTI belonging to phylogenetic groups D and B2. However, phylogenetic groups D and A included UEc isolates that lost genes from the *pap* operon likely during the process of adaptation and immune system evasion. Furthermore, UEc isolates belonging to phylogenetic groups B2 and A were considered pandemic, and MDR isolates were identified as clone O25-ST131.

## Supporting information

S1 Table Primers and PCR conditions used in this study. (PDF)

S2 Table Origin and antibiotic susceptibility profile of UEc isolates from cUTI. (PDF)

S3 Table Origin and antibiotic susceptibility profile of UEc isolates from uUTI. (PDF)
