# Correction: Features of urinary *Escherichia coli* isolated from children with complicated and uncomplicated urinary tract infections in Mexico

**DOI:** 10.1371/journal.pone.0208285

**Published:** 2018-11-26

**Authors:** Víctor M. Luna-Pineda, Sara A. Ochoa, Ariadnna Cruz-Córdova, Vicenta Cázares-Domínguez, Juan P. Reyes-Grajeda, Marco A. Flores-Oropeza, José Arellano-Galindo, Rigoberto Hernández-Castro, Marcos Flores-Encarnación, Adriana Ramírez-Vargas, Héctor J. Flores-García, Leticia Moreno-Fierros, Juan Xicohtencatl-Cortes

The eighth author’s name is spelled incorrectly. The correct name is: Rigoberto Hernández-Castro.
