# Supplementary material for: Features of urinary Escherichia coli isolated from children with complicated and uncomplicated urinary tract infections in Mexico
Source: PLoS One. 2018 Oct 4;13(10):e0204934. doi: 10.1371/journal.pone.0204934 (PMC6171886; doi:10.1371/journal.pone.0204934)
Supplement: S1 Table — (PDF) [file pone.0204934.s001.pdf]

S1 Table. Primers and PCR Conditions used in this Study.

Gene	Primer name	Sequence 5'→3'	Size (pb)	PCR conditions	Reference
		**Determination of the phylogenetic group**			
chuA	F-chuA<br>R-chuA	GACGAACCAACGGTCAGGAT<br>TGCCGCCAGTACCAAAGACA	279	**Pd:** 95°C/5 min/1 cycle<br>**Id:** 95°C/1 min/25 cycles<br>**Al:** 55°C/1 min<br>**Ex:** 72°C/2 min<br>**Fex:** 72°C/5 min<br>**C:** 4°C/∞ min	[16]
yjaA	F-yjaA<br>R-yjaA	TGAAGTGTCAGGAGACGCTG<br>TGGAGAATGCGTTCCTCAAC	211		
*TspE*4.C2	F-TspE4.C2<br>R-TspE4.C2	GAGTAATGTCGGGGCATTCA<br>CGCGCCAACAAAGTATTACG	152		
		**Analysis of virulence and fitness factors**			
hylA	F-hylA<br>R-hylA	CGTGGACACAGCTGCCAGCA<br>TGCAGCGGTGGCGGCATCAT	789	**Pd:** 95°C/5 min/1 cycle<br>**Id:** 95°C/1 min/25 cycles<br>**Al:** 56°C/1 min<br>**Ex:** 72°C/1 min<br>**Fex:** 72°C/5 min<br>**C:** 4°C/∞ min	[57]
iutD	F-iutD<br>R-iutD	TACCGGATTGTCATATGCAGACCGT<br>AATATCTTCCTCCAGTCCGGAGAAG	602		
fimH	F-fimH<br>R-fimH.2	TGCAGAACGGATAAGCCGTGG<br>GCAGTCACCTGCCCTCCGGTA	508		
fyuA	F-fyuA<br>R-fyuA	GCGCATTTGCTGATACTGTTG<br>CATCAGACGATAAGCATGAGCA	272	**Pd:** 95°C/5 min/1 cycle<br>**Id:** 95°C/1 min/30 cycles<br>**Al:** 56°C/30 sec<br>**Ex:** 72°C/1 min<br>**Fex:** 72°C/5 min<br>**C:** 4°C/∞ min	[17,57]
satA	F-satA<br>R-satA	GTTGTCTCTGGCTGTTGC<br>AATGATGTTCCTCCAGAGC	501		
csgA	F-csgA<br>R-csgA	GCCAGTATTTCGCAAGGTGC<br>GGTGTACATATCCCCTTGCTGG	750		
motA	F-motA<br>R-motA	GCGGATTTTTCACCGCACGC<br>GGATCTGCTGGCTCTGC	864	**Pd:** 95°C/2 min/1 cycle<br>**Id:** 94°C/30 sec/30 cycles<br>**Al:** 48°C/1 min<br>**Ex:** 72°C/3 min<br>**Fex:** 72°C/5 min<br>**C:** 4°C/∞	This study
tosA	F-tosA<br>R-tosA	GCACAGCATAACGGGAAAAT<br>CCAGCATGTTACCACGAATG	589		
fliC	fliC.1<br>fliC.2	CCAGTCTGCGCTGTCGAG<br>CACGTTCACGCCGTTGAAC	150		
papF	F-papF<br>R-papF	GTTTTCTGTACCGCTCTCCG<br>CATGCTCATACTGGCCGTG	500	**Pd:** 95°C/2 min/1 cycle<br>**Id:** 95°C/1 min/30 cycles<br>**Al:** 50°C/30 sec<br>**Ex:** 72°C/1 min	This study

Gene	Primer name	Sequence 5'→3'	Size (pb)	PCR conditions	Reference
				**Fex:** 72°C/5 min<br>**C:** 4°C/∞	
papG	F-papGI<br>R-papGI	CAACCTGCTCTCAATCTTTACTG<br>CATGGCTGGTTGTTCCTAAACAT	692	**Pd:** 95°C/2 min/1 cycle<br>**Id:** 94°C/1 min/30 cycles<br>**Al:** 53°C/30 sec<br>**Ex:** 72°C/1 min<br>**Fex:** 72°C/5 min<br>**C:** 4°C/∞	
*papG*I	F-papGII<br>R-papGII	GGAATGTGGTGATTACTCAAAGG<br>TCCAGAGACTGTTCAAGAAGGAC	562		
*papG*II	F-papGIII<br>R-papGIII	CATGGCTGGTTGTTCCTAAACAT<br>TCCAGAGACTGTGCAGAAGGAC	421		
**Clone O25b-ST131**					
O25b	F-O25pabBspe<br>R-O25pabBspe	TCCAGCAGGTGCTGGATCGT<br>GCGAAATTTTTCGCCGTACTGT	347	**Pd:** 95°C/5 min/1 cycle<br>**Id:** 95°C/1 min/25 cycles<br>**Al:** 65°C/1 min<br>**Ex:** 72°C/1.3 min<br>**Fex:** 72°C/5 min<br>**C:** 4°C/∞	[56]
trpA	F-trpA<br>R-trpA	GCTACGAATCTCTGTTTGCC<br>GCAACGCGGCCTGGCGGAAG	427		
**Analysis of the *pap* operon**					
vpapG	F-vpapG<br>R-vpapG	CACAGACTTGTCAGCAGCC<br>GGTTACAGAGTGACAGCAGGTC	1,200	**Pd:** 94°C/2 min/1 cycle<br>**Id:** 94°C/1 min/30 cycles<br>**Al:** 51°C/30 sec<br>**Ex:** 72°C/2.3 min<br>**Fex:** 72°C/5 min<br>**C:** 4°C/∞	This study
papI to *papA*	F-papI<br>R-papA	CAGTGAAGCATGCCCACAG<br>CCGGCAATAACCGACTTAATC	1,182	**Pd:** 94°C/2 min/1 cycle<br>**Id:** 94°C/30 sec/30 cycles<br>**Al:** 49°C/1 min<br>**Ex:** 68°C/3 min<br>**Fex:** 68°C/5 min<br>**C:** 4°C/∞	
papI to *papE*	F-papI<br>R-papE	CAGTGAAGCATGCCCACAG<br>GTCTCAGCATGTACATGCAGC	7,021		
papA to *papE*	F-papA<br>R-papE	GTAGCTATGGCAGTGGTGTC<br>GTCTCAGCATGTACATGCAGC	5,835		
papA to v*papG*	F-papA<br>R-vpapG	GTAGCTATGGCAGTGGTGTC<br>GGTTACAGAGTGACAGCAGGTC	8,055		
**ERIC analysis**					
ERIC	F-ERIC<br>R-ERIC	ATGTAAGCTCCTGGGGATTCAC<br>AAGTAAGTGACTGGGGTGAGCG	Variable	**Pd:** 94°C/5 min/1 cycle<br>25 cycles of the following:<br>**Id:** 94°C/1 min<br>**AI:** 53°C/1 min<br>**Ex:** 72°C/1 min<br>**Fex:**72°C/5 min<br>**C:** 4°C/∞	[26]

Pd: Predenaturation; **Id:** Initial denaturation; **A:** Alignment; **Ex:** Extension; F**ex:** Final extension; **C**: Conservation
