# Supplementary material for: Features of urinary Escherichia coli isolated from children with complicated and uncomplicated urinary tract infections in Mexico
Source: PLoS One. 2018 Oct 4;13(10):e0204934. doi: 10.1371/journal.pone.0204934 (PMC6171886; doi:10.1371/journal.pone.0204934)
Supplement: S2 Table — (PDF) [file pone.0204934.s002.pdf]

## S2 Table. Origin and Antibiotic Susceptibility Profile of UEc Isolates from cUTI.

| Sample number | Cluster | Strains | Ward | Susceptibility Profile | Sample number | Cluster | Strains | Ward | Susceptibility Profile |
|---|---|---|---|---|---|---|---|---|---|
| 1 | I | 370U | NICU | MDR$^{ABCDEG}$ | 62 | VII | 835U-80513 | NEP | R$^{H}$ |
| 2 | II | 190U-210513 | NEP | S | 63 | VII | 684U | EW | MDR$^{ACDEI}$ |
| 3 | II | 734U | NEP | MDR$^{ABCHI}$ | 64 | VII | 883U-190613 | NEP | MDR$^{ABEH}$ |
| 5 | II | 572U-20513 | NEP | R$^{I}$ | 65 | VII | 634U-20513 | INF | MDR$^{ACDEGH}$ |
| 6 | II | 557U1 | NICU | R$^{I}$ | 67 | VII | 204U-180513 | IM | MDR$^{ABCDEGH}$ |
| 8 | III | 208U-30713 | EW | MDR$^{ACDEGH}$ | 68 | VII | 474U-180413 | NEP | R$^{H}$ |
| 9 | III | 207U-30713 | NICU | MDR$^{ABCDEGH}$ | 69 | VII | 525U-180413 | GPS | MDR$^{ABCDE}$ |
| 12 | III | 289U-50613 | NEP | S | 72 | VIII | 638U-240713 | NEP | MDR$^{ABH}$ |
| 14 | IV | 258U-100413 | EW | R$^{AB}$ | 75 | VIII | 496U | GPS | MDR$^{ABCDEG}$ |
| 15 | IV | 990U-140513 | NEP | R$^{H}$ | 76 | IX | 928U-80513 | IM | MDR$^{ABH}$ |
| 16 | IV | 942U-190613 | NEP | MDR$^{ABHI}$ | 77 | IX | 579 U-20513 | NICU | MDR$^{ABH}$ |
| 17 | IV | 361U | NICU | MDR$^{ABCDEGH}$ | 80 | IX | 320U-100413 | EW | MDR$^{ABCDEG}$ |
| 18 | IV | 962U-270613 | NICU | MDR$^{ABH}$ | 81 | IX | 183U-50613 | EW | MDR$^{ABH}$ |
| 19 | IV | 630U-20513 | INF | MDR$^{ABCDEGHI}$ | 83 | IX | 821U | GPS | R$^{AB}$ |
| 20 | IV | 963U-80513 | ONC | R$^{AH}$ | 84 | IX | 251U | EW | MDR$^{ABH}$ |
| 21 | IV | 500U-180413 | GPS | MDR$^{ABCDE}$ | 85 | IX | 561U5 | EW | R$^{AH}$ |
| 22 | IV | 140U-100413 | NEP | MDR$^{ABH}$ | 86 | X | 489U-240713 | EW | MDR$^{ABH}$ |
| 23 | IV | 149U-50613 | NEP | MDR$^{ABCDGHI}$ | 87 | X | 659U-110613 | EW | MDR$^{ABCDEHI}$ |
| 25 | IV | 826U | TE | R$^{AH}$ | 88 | X | 475U-240713 | NEP | MDR$^{ABCDEH}$ |
| 26 | IV | 775U | NEP | MDR$^{ABCDEH}$ | 89 | X | 460U-240713 | NEP | R$^{AE}$ |
| 27 | V | 668U | TE | S | 91 | X | 838U-190613 | NEP | R$^{AB}$ |
| 28 | V | 700U | NEP | MDR$^{ABH}$ | 93 | X | 644U-20513 | EW | MDR$^{ABCDE}$ |
| 29 | V | 536U-240713 | EW | R$^{A}$ | 94 | X | 469U-50613 | ST | MDR$^{ABCDGH}$ |
| 30 | V | 489U-240713 | EW | R$^{AH}$ | 95 | X | 306U-100413 | UR | MDR$^{ABCDEHI}$ |
| 31 | V | 258U | GPS | MDR$^{ABCDEGHI}$ | 96 | XI | 99U-270613 | NEP | S |
| 32 | V | 110U | EW | MDR$^{ABH}$ | 98 | XI | 460U | NEP | R$^{AH}$ |
| 33 | V | 430U | EW | MDR$^{ABCDE}$ | 99 | XI | 47U-270613 | NEP | MDR$^{ABH}$ |
| 34 | V | 244U1-100413 | NEP | MDR$^{ABH}$ | 100 | XI | 642U-110613 | NEP | MDR$^{ABCDEGH}$ |
| 35 | V | 699U | NEP | MDR$^{ABCDEH}$ | 101 | XI | 394U-180413 | NEP | R$^{AH}$ |
| 36 | V | 37U-250313 | EW | R$^{AB}$ | 102 | XI | 964U-200513 | GPS | MDR$^{ABCDEH}$ |
| 37 | V | 430U | EW | MDR$^{ABCDEGHI}$ | 104 | XI | 390U-50613 | EW | R$^{AH}$ |
| 38 | V | 672U | EW | R$^{AB}$ | 106 | XI | 622U-20513 | NEP | MDR$^{ABGH}$ |
| 40 | V | 849U | NEP | MDR$^{ABCDEH}$ | 107 | XI | 873U-190613 | NEP | MDR$^{AEH}$ |
| 43 | V | 100U | HEM | MDR$^{ABCDRGHI}$ | 111 | XI | 158U-100413 | UR | R$^{AH}$ |
| 45 | V | 534U | NEP | S | 113 | XI | 151U | GPS | R$^{AH}$ |
| 46 | V | 742U | NEP | MDR$^{ABH}$ | 117 | XI | 552U | GPS | MDR$^{ABH}$ |
| 50 | VI | 930U-190613 | NEP | MDR$^{ABCDHI}$ | 118 | XI | 422U1 | NEP | R$^{H}$ |
| 51 | VI | 296U-100413 | NEP | MDR$^{ABH}$ | 119 | XII | 325U-100413 | PICU | MDR$^{ABCDEG}$ |
| 52 | VI | 695U-110613 | GPS | MDR$^{ACD}$ | 120 | XII | 974U | NEP | MDR$^{ABH}$ |
| 55 | VII | 249U-50613 | GPS | MDR$^{ABCDEGHI}$ | 122 | XII | 377U | NEP | MDR$^{EHI}$ |
| 56 | VII | 597U-20513 | NEP | R$^{AB}$ | 123 | XII | 920U | NEP | MDR$^{ABCDEH}$ |
| 57 | VII | 9U-140413 | GPS | MDR$^{ABCDEI}$ | 124 | XII | 172U | GPS | R$^{AH}$ |
| 58 | VII | 202U-210513 | IM | MDR$^{ABCDEGHI}$ | 125 | XII | 493U-240713 | HEM | S |
| 59 | VII | 806U-190613 | NEP | MDR$^{ABHI}$ | 127 | XII | 601U-20513 | NEP | MDR$^{AEHDR}$ |
| 61 | VII | 457U-240713 | GPS | S | 131 | XIII | 559U | IT | MDR$^{ABCDEH}$ |
| 133 | XIV | 94U-50613 | NICU | MDR$^{ABH}$ | 153 | XV | 765U | PICU | MDR$^{ABCDEH}$ |
| 134 | XIV | 709U-110613 | TRU | MDR$^{ABH}$ | 157 | XV | 540U | NEP | MDR$^{ABCDEGH}$ |
| 135 | XIV | 694U-110613 | TRU | MDR$^{ABH}$ | 158 | XV | 277U | NICU | MDR$^{ABCDEG}$ |
| 136 | XIV | 94U | GPS | MDR$^{ABCDEGH}$ | 162 | XVI | 336U-240713 | NICU | MDR$^{ABCDEGH}$ |
| 137 | XIV | 998U | NICU | MDR$^{ABH}$ | 163 | XVI | 625U-240713 | NEP | MDR$^{ABGH}$ |
| 139 | XIV | 799U | UR | MDR$^{ABCDEGH}$ | 164 | XVI | 673U-110613 | NEP | R$^{E}$ |
| 140 | XIV | 769U | GPS | MDR$^{ABCDEG}$ | 165 | XVI | 831U | NEP | R$^{AE}$ |
| 143 | XV | 320U-100713 | NICU | MDR$^{ABCDEGH}$ | 166 | XVI | 785U | IT | R$^{AE}$ |

| Sample number | Cluster | Strains | Ward | Susceptibility Profile | Sample number | Cluster | Strains | Ward | Susceptibility Profile |
|---|---|---|---|---|---|---|---|---|---|
| 144 | XV | 336U-100713 | NICU | $MDR^{ABCDEGH}$ | 167 | XVI | 986U | NEP | $R^{AH}$ |
| 145 | XV | 972U-270613 | UR | $MDR^{ABCDEH}$ | 168 | XVI | 524U-240713 | NEP | $MDR^{ABCDEG}$ |
| 146 | XV | 320U-240713 | NICU | $MDR^{ABCDEGH}$ | 169 | XVI | 614U-50613 | NEP | $MDR^{ABCDEH}$ |
| 148 | XV | 625U4-240713 | NEP | $MDR^{ABGH}$ | 170 | XVI | 244U-30713 | NEP | $R^{EH}$ |
| 149 | XV | 752U-20513 | NEP | $MDR^{AEH}$ | 172 | XVI | 673U-110613 | NEP | $R^{E}$ |
| 150 | XV | 250U-100413 | IM | $MDR^{ABCDEGHI}$ | 176 | XVI | 920U-80513 | NEP | $MDR^{AEH}$ |
| 152 | XV | 787U-20513 | IM | $MDR^{ABCDEGHI}$ | 177 | XVI | 202U-30713 | NICU | $MDR^{ABCDEG}$ |

Neonatal intensive care unit (NICU), Nephrology (NP), Emergency ward (EW), Oncology (ONC), Tissues engineering (TE), Hematology (HEM), Allergies (AL), Internal medicine (IM), Urology (UR), Pediatric intensive care unit (PICU), Internal Therapy (IT), Transplants unit (TRU), Infectology (INF), General pediatric surgery (GPS), Penicillins (A), β-lactam/β-lactamase inhibitor combinations (B), Cephems-2$^{nd}$ generation (C), Cephems-3$^{rd}$ generation (D), Fluoroquinolones (E), Carbapenems (F), Aminoglycosides (G), Folate pathway inhibitors (H), Nitrofurans (I), Sensitive to all antibiotics (S), Resistant (R)
