# Supplementary material for: Features of urinary Escherichia coli isolated from children with complicated and uncomplicated urinary tract infections in Mexico
Source: PLoS One. 2018 Oct 4;13(10):e0204934. doi: 10.1371/journal.pone.0204934 (PMC6171886; doi:10.1371/journal.pone.0204934)
Supplement: S3 Table — (PDF) [file pone.0204934.s003.pdf]

S3 Table. Origin and Antibiotic Susceptibility Profile of UEc Isolates from uUTI.

Sample number	Cluster	Strains	Ward	Susceptibility Profile
4	II	770U	GA	MDR^{ABH}
7	II	532U1	EC	MDR^{ABCDEH}
10	III	486U-240713	CLW	R^{AH}
11	III	665U-110613	CLW	S
13	IV	400U-240713	CLW	MDR^{ABGH}
24	IV	591U	CLW	MDR^{ABCDEGHI}
39	V	608U	NEU	MDR^{ABCDEGH}
41	V	966U	EC	S
42	V	287U	CLW	MDR^{ABH}
44	V	584U	CLW	S
47	V	740U	PED	R^{AB}
48	VI	856U-190613	PED	R^{AH}
49	VI	13U-140513	AL	S
53	VI	944U	CS	R^{AH}
54	VII	444U-240713	GA	S
60	VII	670U-200513	EC	MDR^{ABCDEGH}
70	VII	523U	AL	MDR^{ABCDEH}
71	VII	440U1	EC	R^{AH}
73	VIII	385U	CLW	R^{A}
74	VIII	653U-110613	CLW	MDR^{ABCDH}
78	IX	326U-240513	CLW	MDR^{ABH}
79	IX	245U-100413	NS	R^{AH}
82	IX	40U-100413	EC	MDR^{ABCDH}
90	X	857U-190613	GA	MDR^{ACDGH}
92	X	646U-110613	EC	MDR^{ABDHI}
97	XI	363U-100413	EC	MDR^{ABH}
103	XI	227U-100413	NS	R^{AH}
105	XI	256U	CLW	R^{AB}
108	XI	775U-20513	CLW	R^{AH}
109	XI	303U-100413	NS	R^{AH}
110	XI	864U-250313	EC	MDR^{ABCH}
112	XI	564U-20513	AD	R^{AH}
114	XI	589U	EC	MDR^{ABH}
115	XI	897U-190613	GA	MDR^{ABH}
116	XI	644U-110613	GA	MDR^{ABH}
121	XII	630U-240713	EC	S
126	XII	213U-100413	CLW	S
128	XII	532U5C4	EC	MDR^{ABCDE}
129	XIII	943U	CLW	MDR^{ABHI}
130	XIII	742U	PED	S
132	XIII	542U	CLW	S
138	XIV	943U	CLW	MDR^{ABHI}
141	XIV	565U	CLW	MDR^{ABCDEG}
142	XV	333U-100413	GA	MDR^{ABCDEG}
147	XV	400U-100713	CLW	MDR^{ABGH}
151	XV	732U-20513	CLW	MDR^{ABCDEG}
154	XV	487U	CLW	MDR^{ABCDEG}
155	XV	608U-110613	NEU	MDR^{ABCDEH}
156	XV	50U-50613	NEU	MDR^{ABCDEH}
159	XV	324U-220513	AD	MDR^{ABCDEGH}
160	XV	425U	GA	MDR^{ABCDEG}
161	XVI	400U1-240713	CLW	MDR^{ABCDGH}
171	XVI	951U	EC	MDR^{ABCH}
173	XVI	561U-50613	CLW	R^{AH}
174	XVI	387U-50613	CLW	MDR^{ACDEG}
175	XVI	942U-80513	CLW	MDR^{AEH}
178	XVI	177U3	EC	MDR^{ABCDEG}

Gastroenterology (GA), Classification Ward (CLW), Neurology (NEU), Allergies (AL), Children's stay (CS), Adolescent (AD), External Consultation (EC), Penicillins (A), β-lactam/β-lactamase inhibitor Combinations (B), Cephems-2nd generation (C), Cephems-3rd generation (D), Fluoroquinolones (E), Carbapenems (F), Aminoglycosides (G), Folate pathway inhibitor (NH), Nitrofurans (I), Sensitive to All Antibiotics (S), Resistant (R).
